# Phthalates impact on the epigenetic factors contributed specifically by the father at fertilization

**DOI:** 10.1186/s13072-022-00475-2

**Published:** 2023-01-24

**Authors:** G. M. Swanson, F. L. Nassan, J. B. Ford, R. Hauser, J. R. Pilsner, S. A. Krawetz

**Affiliations:** 1 Department of Obstetrics & Gynecology, Wayne State University School of Medicine, 275 E. Hancock, Detroit, MI 48201 USA; 2 Center for Molecular Medicine & Genetics, Wayne State University School of Medicine, 275 E. Hancock, Detroit, MI 48201 USA; 3 Biogen, Cambridge, MA USA; 4 Departments of Environmental Health and Epidemiology, Harvard T. H. Chan School of Public Health, Boston, MA USA; 5 Departments of Environmental Health, Harvard T. H. Chan School of Public Health, Boston, MA USA; 6 Institute of Environmental Health Sciences, Wayne State University, Detroit, MI USA

**Keywords:** Phthalates, Sperm RNA, Paternal contribution, Chromatin modifiers

## Abstract

**Background:**

Preconception exposure to phthalates such as the anti-androgenic dibutyl-phthalate (DBP) impacts both male and female reproduction, yet how this occurs largely remains unknown. Previously we defined a series of RNAs expressly provided by sperm at fertilization and separately, and in parallel, those that responded to high DBP exposure. Utilizing both populations of RNAs, we now begin to unravel the impact of high-DBP exposure on those RNAs specifically delivered by the father.

**Results:**

Enrichment of RNAs altered by DBP exposure within the Molecular Signature Database highlighted cellular stress, cell cycle, apoptosis, DNA damage response, and gene regulation pathways. Overlap within each of these five pathways identified those RNAs that were specifically (≥ fivefold enriched) or primarily (≥ twofold enriched) provided as part of the paternal contribution compared to the oocyte at fertilization. Key RNAs consistently altered by DBP, including *CAMTA2* and *PSME4,* were delivered by sperm reflective of these pathways. The majority (64/103) of overlapping enriched gene sets were related to gene regulation. Many of these RNAs (45 RNAs) corresponded to key interconnected CRREWs (Chromatin remodeler cofactors, RNA interactors, Readers, Erasers, and Writers). Modeling suggests that *CUL2*, *PHF10,* and *SMARCC1* may coordinate and mechanistically modulate the phthalate response.

**Conclusions:**

Mediated through a CRREW regulatory network, the cell responded to exposure presenting stressed-induced changes in the cell cycle—DNA damage—apoptosis. Interestingly, the majority of these DBP-responsive epigenetic mediators’ direct acetylation or deacetylation, impacting the sperm's cargo delivered at fertilization and that of the embryo.

**Supplementary Information:**

The online version contains supplementary material available at 10.1186/s13072-022-00475-2.

## Background

It is now well-known that sperm delivers an entire set of extra-chromosomal components, including RNAs, at fertilization ([1], reviewed in [2]). We defined a series of paternal-provided RNA elements (REs, exon-sized sequences) that are enriched at least fivefold above the oocyte and delivered at fertilization, providing a unique set of RE-containing RNAs (RE-RNAs) while markedly enhancing those present in the oocyte [3]. To date, several of these sperm RE-RNAs have been shown to respond to exposures reflective of lifestyle [4–7]. Some have now been implicated in offspring phenotype [6, 8–11], highlighting the importance of understanding their role in development.

Phthalates are endocrine disruptors widely used in consumer products [5, 12–14], such as vinyl plastics, personal care products, and some medication coatings [15–18]. To date, it is known that phthalate exposure in males has an adverse impact on semen and embryo quality, as well as time to pregnancy [19–22], but the mechanism(s) remains obscure. To begin to address this gap, we defined a series of sperm REs that respond to phthalates [5] using the dibutyl-phthalate (DBP) Inflammatory Bowel Disease (IBD) mesalamine crossover cross-back model (reviewed in [17]). To evaluate the impact of high-DBP exposure, we recruited men taking one of two formulations of mesalamine; one was encapsulated in a DBP-containing coating, and one was without DBP in the coating. Sperm RE-RNAs [5] were isolated and compared to those observed in the oocyte and zygote. Two paternal provided classes were defined from REs present in the zygote. Those paternal provided REs enriched ≥ fivefold compared to the oocyte [3], and excluding those defined here as fivefold enriched [3], those paternal REs, ≥ twofold enriched compared to the oocyte. Through RE expression, we show the mechanistic impact of high-DBP exposure acting through epigenetic modifiers and how they affect those RE-RNAs paternally provided to the oocyte at fertilization.

## Results

Sperm RNAs are known to respond to environmental exposures [4–6]-like dibutyl-phthalate (DBP), an endocrine disruptor found in some medications, including the coating of Asacol, whose active ingredient is mesalamine used to treat Inflammatory Bowel Disease (IBD) [5, 15, 22]. At the recommended maximal Asacol dosage, DBP exposure from the coating exceeds the Environmental Protection Agency (EPA) reference dose for a 150-pound individual by 300–700% (reviewed in [5, 23]) based on the DBP primary urinary metabolite, monobutyl phthalate (MBP). Men on Asacol had MBP urinary concentrations 1,000 times higher than the median male concentration reported in the National Health and Nutrition Examination Survey (NHANES [24] within the general United States population [23]. However, studies also indicate that lower level environmental background exposures to DBP from personal care and consumer products may impact semen and embryo quality, and time to pregnancy [19–22]. Analysis of those sperm RNA Elements (REs, exon-sized RNA fragments) altered in response to high-DBP exposure from using DBP-coated mesalamine, Asacol, compared to the non-DBP coated mesalamine, Pentasa, has begun to define DBP exposome pathways that impact sperm RE-containing RNAs (RE-RNAs) [5].

**Fig. 1:**
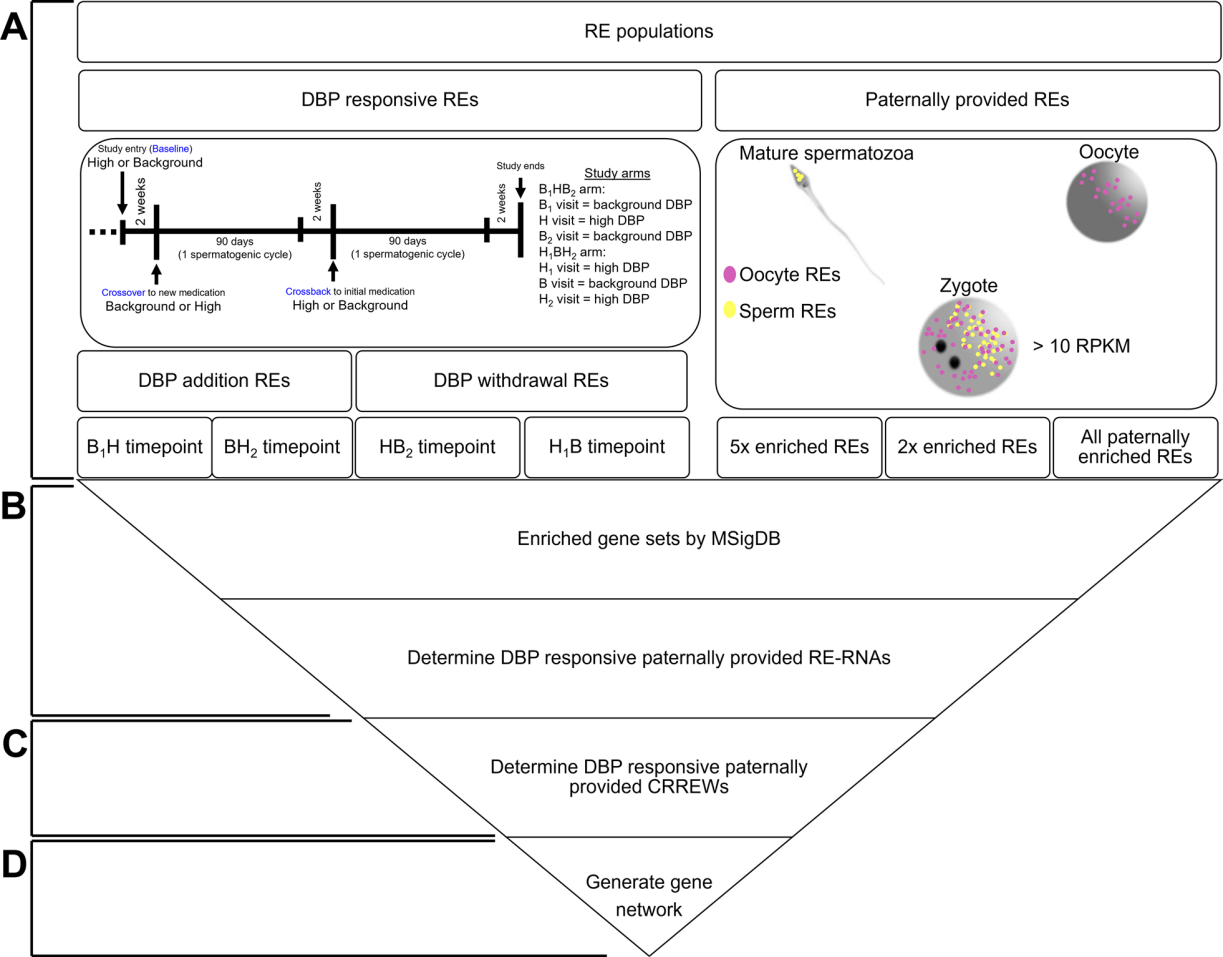
Study analysis design. **A** Briefly, dibutyl-phthalate (DBP) responsive RNA Element (RE)-containing RNAs (RE-RNAs) from each crossover–crossback segment and paternally provided set were identified. **B** Ontology was assessed by the Molecular Signature Database (MSigDB), and shared DBP responsive and paternally provided RE-RNAs determined. **C** Shared RE-RNAs were evaluated for presence in overlapping DBP responsive and paternally provided MSigDB enriched gene sets. CRREWs (chromatin remodeler cofactors, RNA interactors, Readers, Erasers, and Writers) RE-RNAs were identified, and their presence within overlapping DBP responsive and paternally provided MSigDB gene sets was assessed. **D** Gene network to visualize CRREW biological process interactions was generated. H_1_B; high-DBP (baseline visit) to background DBP (crossover visit), BH_2_; background DBP (crossover visit) to high-DBP (crossback visit), B_1_H; background DBP (baseline visit) to high-DBP (crossover visit), HB_2_; high-DBP (crossover visit) to background DBP (crossback visit)

As summarized in Fig. 1A, REs responsive to high-DBP exposure [5] modifying the male contribution at fertilization was considered. Men who were on non-DBP coated mesalamine (e.g., Pentasa) transitioned to high-DBP coated mesalamine (Asacol) (referred to as baseline to crossover; B_1_H) in the baseline non-DBP (B_1_HB_2_) study arm as well as men transitioning from non-DBP coated mesalamine (e.g., Pentasa) back to high-DBP coated mesalamine (Asacol) (referred to as crossover to crossback; BH_2_) in the high-DBP (H_1_BH_2_) study arm, were considered. Comparison of REs responding to DBP withdrawal was from the men starting on high-DBP coated mesalamine transitioning to non-DBP coated mesalamine (baseline to crossover; H_1_B) and men transitioning from high-DBP-coated mesalamine back to non-DBP-coated mesalamine (crossover to crossback; HB_2_).

### Biological pathways altered by DBP exposure

**Fig. 2:**
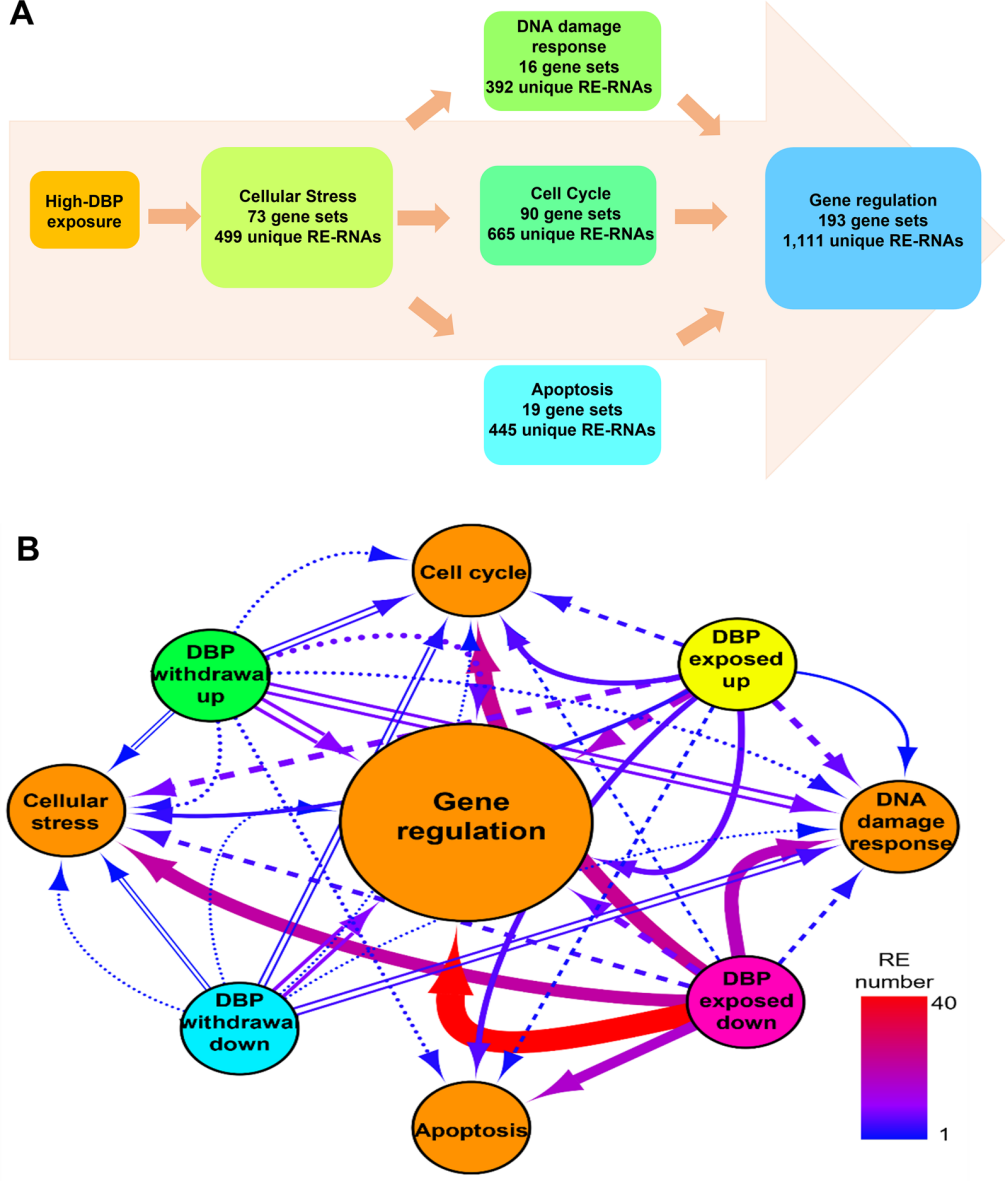
Biological pathways enriched in response to dibutyl-phthalate (DBP). **A** DBP responsive enriched biological pathways were identified using the Molecular Signature Database (MSigDB). The total number of unique RE-RNAs and enriched gene sets is provided. Enriched gene sets were either unique to DBP study arm (B_1_HB_2_ or H_1_BH_2_) or within each study arm. B_1_HB_2_ provided the background DBP study arm baseline to crossover to crossback, H_1_BH_2_ provided the high-DBP study arm baseline to crossover to crossback. **B** DBP-responsive RNA Elements (REs) within the enriched biological pathways that share paternally provided RE-containing RNAs (RE-RNA) were identified. Paternally provided: REs determined as fivefold paternally enriched or twofold paternally enriched. Node color indicates the following; *Orange*: enriched biological processes, *Yellow*: RE abundance increases following DBP exposure, *Pink*: RE abundance decreases upon DBP exposure, *Green*: RE abundance increases upon DBP withdrawal, *Blue*: RE abundance decreases upon DBP withdrawal. Edge color indicates the total number of DBP responsive REs (1–40 REs) with an overlapping paternally provided RE-associated gene name moving between nodes. Scaling for edge color is continuous with 1 RE attributed to a *Dark Blue* color and 40 REs attributed to a *Red* color. Edge line type indicates the following; *solid*: background DBP (baseline visit) to high-DBP (crossover visit) (B_1_H), *parallel lines*: high-DBP (crossover visit) to background DBP (crossback visit) (HB_2_), *dash*: high-DBP (baseline visit) to background DBP (crossover visit) (H_1_B), *dots*: background DBP (crossover visit) to high-DBP (crossback visit) (BH_2_)

The REs defined from this series of high-DBP exposures or withdrawals were associated with biological pathways within the Molecular Signature Database (MSigDB) using the DBP responsive RE-RNAs summarized in Fig. 1B (Additional file 2: Table S1) as input. These included all positively or negatively correlated RE-RNAs, or the entire set of correlated RE-RNAs described as altered between each study visit, e.g., B_1_H (high-DBP exposure). For each set of genes, enrichment (Fig. 1B) identified five major biological processes; cellular stress, cell cycle, DNA damage response, apoptosis, and gene regulation (Fig. 2A, Additional file 3: Table S2). Each enriched biological process included specific MSigDB gene sets within both study arms (B_1_HB_2_ and H_1_BH_2_).

The majority of enriched gene sets were associated with gene regulation (191/392 gene sets), indicating a large proportion of DBP-responsive RE-RNAs function as either transcription factors (TFs) or CRREWs (Chromatin remodeler cofactors, RNA interactors, Readers, Erasers, and Writers [4]). DBP-responsive TF binding sites were identified, and the number of unique TFs assigned to these binding sites is summarized in Additional file 4: Table S3A. However, there was no statistical significance in the number DBP responsive TF encoded RE-RNAs (Additional file 4: Table S3A), which suggested other modulators of gene expression may dominate, and CRREWs were considered (Fig. 1B and D, Additional file 5: Table S4). The proportion of CRREWs was higher than expected (Additional file 4: Table S3B). Accordingly, their potential as modulators among the various enriched biological processes was examined (Fig. 2A). In total, 119 CRREWs were specific to the analysis within the B_1_HB_2_ comparisons, while 60 CRREWs were specific to those within the H_1_BH_2_ comparisons. Fifty CRREWs were represented in the analysis of both study arms (Additional file 5: Table S4). Together this suggests that a start arm's initial drug coating (i.e., high-DBP coated mesalamine or non-DBP coated mesalamine) impacts these biological processes through a unique set of CRREW modulators. In total, 50 DBP-responsive CRREWs were shared between both study arms modulating these biological processes.

### DBP-responsive and paternally provided RE-RNAs

Consideration was given to whether the DBP-responsive RE-RNAs and enriched pathways were present, enriched, and/or unique in those provided by the father at fertilization. Enriched vs unique paternal REs were defined by the presence, or lack of the RE in the oocyte, with an RPKM < 2 considered absent, as this is the lower threshold in which RE presence exceeds experimental error. Two types of paternally provided REs, and their associated gene names (RE-RNAs), were examined. The first comprised the 289 REs (from 206 RE-RNAs) previously defined as paternally enriched (fivefold paternally enriched, Fig. 1A) [3] from a non-IBD population exposed only to background levels of DBP. The second included an additional set of 250 REs (from 93 RE-RNAs), not including those previously defined, that appear to have at least a twofold enrichment in sperm compared to the oocyte (twofold paternally enriched, Fig. 1A, Additional file 6: Table S5). Both paternally provided sets were identified from those observed at a level of at least 10 RPKM in the zygote. A total of 83 REs (5 × paternally enriched: 75 REs [3], 2 × paternally enriched: 8 REs (Additional file 6: Table S5)) were specific to the sperm. In addition, as previously stated, an RPKM > 10 in the zygote was a requirement for the paternally provided REs. This leads to the possibility of the RPKM abundance in the zygote exceeding the combined RPKM in the sperm and oocyte. Only 2% of the paternally provided RE-RNAs (11/539 RE-RNAs) > 5 RPKM were more abundant in the zygote than in sperm. Comparing these paternally provided RE-RNAs to those defined as DBP responsive defined a series of RE-RNAs that were both DBP responsive and paternally provided (Figs. 1B, 2B).

Using Cytoscape [25] to aid visualization, the 132 paternally provided DBP-responsive RE-RNAs identified in Fig. 2B represented a significantly larger overlap between data sets than expected (*p* < 4.358e-35, representation factor = 3.0). Of these RE-RNAs, a significant proportion was CRREWs (45 CRREWs; Fig. 1C, Table 1, Additional file 7: Table S6). From this list, 86 RE-RNAs, including 38 CRREWs (Table 1), were within the DBP-enriched biological processes, including *GOMF chromatin binding* and *GOBP cellular response to stress* (Figs. 1B, 2B). Most of the DBP responsive CRREWs were specific to the B_1_HB_2_ or the H_1_BH_2_ comparisons. The chromatin remodeler cofactors *PHF10*, *CUL2*, *ATXN7*, *PHC3*, *SMARCC1,* and *EYA3,* were identified (Table 1), indicating these CRREWs likely modulate the DBP response between these processes. Of these, *CUL2*, *PHF10,* and *SMARCC1* were visually full-length in all DBP responsive and paternally provided samples that passed the Transcript Integrity Index (TII) for identification of samples of similar RNA quality [26] (Table 1, Additional file 1: Figure S1). Their representation in the shared enriched gene sets within each biological process supports the essential role of these three CRREWs in the DBP response.

**Table 1:** Dibutyl-phthalate (DBP) responsive Chromatin remodeler cofactors, RNA interactors, Readers, Erasers, and Writers (CRREWs)

A. fivefold Paternal enriched and DBP responsive CRREWs												
CRREW	CRREW class	Human sperm proteome	Paternally provided samples			DBP responsive samples						
			Visually full-length	RE	Mean abundance	DBP study arm	Visually full-length	RE	Baseline mean abundance	Crossover mean abundance	Crossback mean abundance	Movement with phthalate
**CUL2**	**Chromatin remodeler cofactor**	**Yes**	**Full-length**	**chr10_35049683_35049765**	**73.34378112**	**B**_**1**_**HB**_**2**_	**Full-length**	**chr10_35071199_35071339**^a^	**45.88953277**	**36.94494826**	**33.3796977**	**Down—same**
				**chr10_35054434_35054539**	**90.27970798**							
				**chr10_35060874_35060968**	**124.36174959**	**H**_**1**_**BH**_**2**_	**Full-length**	***chr10_35031300_35031386***	**10.9300058**	**12.60999224**	**16.5514561**	**Up—up**
				**chr10_35062960_35063062**	**76.39002731**							
EFCAB6	Chromatin remodeler cofactor	Yes	Full-length	chr22_43755766_43755832	47.99058386	H_1_BH_2_	Full-length	chr22_43808995_43809131^b^	23.04421341	22.47121388	30.92004033	Same—up
FBRS	Chromatin remodeler cofactor	No	Full-length	chr16_30661180_30661215	139.16671978	H_1_BH_2_	Full-length	chr16_30665035_30665079^a^	11.7002433	23.79308874	13.77795832	Up—same^c^
				chr16_30661304_30661333	163.82992422							
				chr16_30662420_30662468	322.18875278			chr16_30665306_30665401^a^	15.91554696	33.45144483	17.72288399	Up—same^c^
				chr16_30668772_30668979	38.16171231			chr16_30665411_30665530^b^	25.49395297	12.03010593	22.8260116	Same -up^c^
FUS	RNA interactor	Yes	Full-length	chr16_31189665_31189794	90.62018963	B_1_HB_2_	Full-length	chr16_31179681_31179790^a^	17.54623506	8.059807977	9.112771891	Down -same
**PHF10**	**Chromatin remodeler cofactor**	**No**	**Full-length**	**chr6_169703905_169704088**	**56.77073068**	**B**_**1**_**HB**_**2**_	**Full-length**	**chr6_169712386_169712539**^a^	**104.6714394**	**73.28088898**	**72.69274424**	**Down—same**^c^
								**chr6_169717742_169717906**^b^	**26.5058166**	**21.75793957**	**28.02153713**	**Same -up**^c^
						**H**_**1**_**BH**_**2**_	**Full-length**	**chr6_169715708_169715857**^b^	**46.4094406**	**41.91086488**	**25.73117816**	**Same—down**^c^
								**chr6_169715266_169715305**^b^	**9.913662168**	**3.92151334**	**12.24840542**	**Same—up**^c^
TP53BP1	Reader	No	Full-length	chr15_43438324_43438416	60.10469689	B_1_HB_2_	Full-length (≥ 1 < 35 samples)	chr15_43491669_43491753^a^	90.52982404	57.56060158	54.69346007	Down—same^c^
								chr15_43474673_43474767^a^	43.09581869	24.32817473	22.95757362	Down—same^c^
								chr15_43477593_43477759^b^	19.25400217	11.49681526	12.46340685	Same—up^c^

B. twofold Paternally enriched and DBP responsive CRREWs												
CRREW	CRREW class	Human sperm proteome	Paternally provided samples			DBP responsive samples						
			Visually full-length	RE	Mean abundance	DBP study arm	Visually full-length	RE	Baseline mean abundance	Crossover mean abundance	Crossback mean abundance	Movement with phthalate
ACTB	Chromatin remodeling cofactor	No	Full-length (≥ 1 < 7 samples)	chr7_5529161_5529400	608.1603759	B_1_HB_2_	Fail	chr7_5527147_5527891^a^	9.927226458	39.23923731	76.15182438	Up—same
				chr7_5529535_5529663	160.4580747							
ASXL3	Chromatin remodeling cofactor	Yes	Full-length (≥ 1 < 7 samples)	chr18_33607594_33607676^d^	126.69565505	H1BH2	Fail	chr18_33605537_33605658^b^	49.02305219	57.68603612	29.20425494	Same—down
								chr18_33607594_33607676^b^^, d^	100.1024646	122.0676208	65.30310662	
**ATXN7**	**Chromatin remodeling cofactor**	**No**	**Full-length**	**chr3_63913157_63913225**	**23.23374838**	**B1HB2**	**Full-length (≥ 1 < 35 samples)**	**chr3_63971410_63971449**^a^	**44.13861655**	**18.85341672**	**31.88951213**	**Down—up**^c^
				**chr3_63952379_63952483**	**65.56893528**			**chr3_63970990_63971199**^a^	**28.29537781**	**16.13056589**	**15.78642347**	**Down—same**^c^
				**chr3_63979915_63980167**	**65.82895373**			**chr3_63970590_63970729**^a^	**28.9651738**	**31.0349008**	**22.84394278**	
				**chr3_63982186_63982445**	**51.9743582**							
				**chr3_63982939_63983021**	**86.70154882**	**H1BH2**	**Full-length (≥ 1 < 55 samples)**	**chr3_63990176_63990374**^a^	**15.20540033**	**23.06433484**	**18.39674764**	**Up—same**
				**chr3_63988059_63988324**	**86.65469577**							
				**chr3_63997621_63997687**	**20.627258**							
BRD2	Reader	No	Full-length (≥ 1 < 7 samples)	chr6_32974462_32974765^d^	167.2971302	B1HB2	Full-length (≥ 1 < 35 samples)	chr6_32974462_32974765^a^^, d^	148.0520041	95.59297034	97.45927523	Down—same
				chr6_32975384_32975521	244.581243							
				chr6_32976031_32976169	246.1430444							
				chr6_32976250_32976464	449.0591325							
				chr6_32978126_32978388	290.1415452			chr6_32975021_32975112^a^	88.41784757	53.73315186	53.689177	
				chr6_32979828_32980132	215.4520379							
				chr6_32980342_32980464	246.6401318							
CBX8	Reader	No	Full-length (≥ 1 < 7 samples)	chr17_79796250_79796315	109.1088811	B1HB2	Full-length (≥ 1 < 35 samples)	chr17_79796497_79796540^a^	67.72774064	35.94307833	47.37031239	Down—same
CUL4A	Writer	Yes	Full-length (≥ 1 < 7 samples)	chr13_113245152_113245237^d^	45.83401639	H1BH2	Fail	chr13_113245152_113245237^b^^, d^	4.749607197	11.83695093	4.810742772	Same—down
DDX21	RNA interactor	No	Full-length (≥ 1 < 7 samples)	chr10_68956077_68956312	45.59930818	B1HB2	Full-length (≥ 1 < 35 samples)	chr10_68973545_68973664^a^	12.48308777	6.652350194	6.801472421	Down—same
				chr10_68956128_68956312	58.23824687							
				chr10_68959806_68960249	27.30981821							
DDX5	Chromatin remodeling cofactor	No	Full-length	chr17_64499616_64500326	50.18733594	H1BH2	Full-length (≥ 1 < 55 samples)	chr17_64508057_64508199^a^	18.07129807	31.49088399	21.44690173	Up—same
				chr17_64502439_64502549	222.7743864							
				chr17_64502926_64503098	174.5232727							
				chr17_64504677_64504842	562.365823							
**EYA3**	**Eraser**	**No**	**Full-length (≥ 1 < 7 samples)**	**chr1_28035544_28035680**	**40.65905966**	**B1HB2**	**Full-length (≥ 1 < 35 samples)**	**chr1_27989697_27989811**^a^	**17.51387147**	**8.11829011**	**7.684114697**	**Down—same**
				**chr1_28038839_28038905**	**71.9858758**							
				**chr1_28042571_28042650**^d^	**82.19180154**							
				**chr1_28048383_28048426**	**55.11261243**	**H1BH2**	**Full-length (≥ 1 < 55 samples)**	**chr1_28042571_28042650**^b^^, d^	**89.90445658**	**77.74204547**	**93.81864264**	**Same—up**
				**chr1_28057994_28058094**	**55.29632114**							
FBL	Writer	No	Full-length (≥ 1 < 7 samples)	chr19_39834668_39834813	60.41931957	H1BH2	Fail	chr19_39839859_39839958^b^	18.59527254	27.37708055	9.213151401	Same—down
				chr19_39840233_39840327	50.69038934							
FOXP1	Chromatin remodeling cofactor	No	Full-length (≥ 1 < 7 samples)	chr3_71041328_71041532^d^	42.11617683	H1BH2	Fail	chr3_71041328_71041532^b^^, d^	15.6839618	13.71369054	15.54280611	Same—up
FOXP4	Chromatin remodeling cofactor	No	Fail	chr6_41587793_41587897	39.27112581	B1HB2	Fail	chr6_41591221_41591322^a^	45.87619906	26.85038148	20.82985849	Down—same
				chr6_41597176_41597242	144.390806							
FTO	Eraser	No	Fail	chr16_53873786_53873865	21.75113943	B1HB2	Fail	chr16_54055231_54055250^a^	69.62113525	20.61269425	18.47722558	down—same
GLYR1	Reader	No	Fail	chr16_4822875_4822931	85.08743926	H1BH2	Fail	chr16_4845074_4845153^b^^, d^	36.46972236	46.9269976	20.87481521	Same—down
				chr16_4832774_4832912	56.47345151			chr16_4846174_4846210^b^^, d^	67.90414093	142.7455894	36.28550649	
				chr16_4845074_4845153^d^	86.16449545			chr16_4847228_4847342^b^	18.02496292	35.7298856	10.55306118	
				chr16_4846174_4846210^d^	214.7925019							
GSK3B	Writer	No	Full-length	chr3_119863419_119863605	47.57577249	B1HB2	Full-length (≥ 1 < 35 samples)	chr3_119947268_119947351^a^^, d^	9.469777621	4.330235615	3.812284788	Down—same
				chr3_119876413_119876508	74.65344499							
				chr3_119905755_119905852	40.17183664							
				chr3_119947268_119947351^d^	8.201198964							
HDGF	Chromatin remodeling cofactor	No	Full-length (≥ 1 < 7 samples)	chr1_156742107_156743455	29.31885782	B1HB2	Full-length (≥ 1 < 35 samples)	chr1_156746941_156747070^a^	38.46470389	21.80162743	33.42011021	Down—up
				chr1_156745008_156745146	103.5140225							
				chr1_156745297_156745373	153.7851866							
JAK2	Writer	No	Fail	chr9_5044403_5044520	73.43333397	B1HB2	Fail	chr9_5093247_5093296^a^	20.58490527	32.01820286	32.69512962	Up—same
				chr9_5050686_5050831	107.4942939							
				chr9_5054563_5054884	130.4274597							
				chr9_5064883_5065040	25.21798197							
				chr9_5066678_5066789	24.52971222							
				chr9_5069022_5069208	21.28614607							
				chr9_5072492_5072626	14.53980654							
JMJD1C	Eraser	No	Full-length	chr10_63215263_63215455	35.51650661	B1HB2	Full-length (≥ 1 < 35 samples)	chr10_63380318_63380482^b^	17.4600717	19.05969651	18.90688543	Same—up
KDM3B	Eraser	No	Full-length (≥ 1 < 7 samples)	chr5_138381516_138381590	46.44162202	B1HB2	Fail	chr5_138381516_138381748^a^	12.66998754	4.873031368	7.239254403	Down—same
METTL14	Writer	No	Full-length	chr4_118689370_118689457	123.1705451	B1HB2	Full-length (≥ 1 < 35 samples)	chr4_118694436_118694526^a^^, d^	83.51980054	46.03741119	51.4908201	Down—same
				chr4_118691981_118692068	31.29652938							
				chr4_118694436_118694526^d^	64.94446923							
				chr4_118703935_118704051	25.19397513							
NCL	Chromatin remodeling cofactor	No	Full-length (≥ 1 < 7 samples)	chr2_231455378_231455397	102.5438988	B1HB2	Fail	chr2_231461540_231462017^a^	5.236450253	20.29187093	12.3903226	Up—same
				chr2_231455401_231455624	67.46658314							
PBK	Writer	No	Fail	chr8_27833056_27833133	84.32665353	B1HB2	Fail	chr8_27822319_27822488^a^	29.13220667	13.70207793	20.37717724	Down—same
**PHC3**	**Chromatin remodeling cofactor**	**No**	**Full-length (≥ 1 < 7 samples)**	**chr3_170136419_170136665**	**47.04021032**	**B1HB2**	**Fail**	**chr3_170136366_170136670**^b^	**33.10320015**	**23.37991969**	**38.36697809**	**Same—up**^c^
								**chr3_170125959_170126008**^b^^, e^	**50.56724341**	**47.14446851**	**39.30243571**	**Same—down**^c^
				**chr3_170145423_170145521**	**43.83520446**	**H1BH2**	**Full-length (≥ 1 < 55 samples)**	**chr3_170125959_170126008**^b^^, e^	**52.42505534**	**68.67472239**	**68.63626944**	**Up—same**
PIAS2	Chromatin remodeling cofactor	Yes	Full-length (≥ 1 < 7 samples)	chr18_46829734_46829867	126.9338048	H1BH2	Full-length (≥ 1 < 55 samples)	chr18_46864164_46864248^b^	22.18313576	34.11708408	15.93112434	Same—down
				chr18_46890580_46891054	94.25493752							
PKM	Writer	Yes	Full-length	chr15_72199029_72199756	209.0372187	H1BH2	Full-length (≥ 1 < 55 samples)	chr15_72218943_72219110^b^	227.6530273	228.3467372	257.298983	Same—up
				chr15_72202454_72202620	90.41567139							
				chr15_72203022_72203188	104.69183							
				chr15_72206728_72206880	186.5303306							
				chr15_72207127_72207277	114.0294777							
				chr15_72208621_72208891	435.9339603			chr15_72221168_72221284^b^	509.0236626	472.2128562	505.8016084	
				chr15_72209673_72209859	216.5984929							
				chr15_72210347_72210478	202.6510004							
				chr15_72217409_72217500	179.5251466							
				chr15_72218944_72219110	477.2075803							
PRDM2	Writer	No	Full-length (≥ 1 < 7 samples)	chr1_13731000_13731117	24.97864201	B1HB2	Fail	chr1_13732779_13732882^a^^, d^	18.88791629	10.83481881	12.63840862	Down—same
				chr1_13732779_13732882^d^	37.83172965							
RAD23B	Chromatin remodeling cofactor	No	Full-length	chr9_107300141_107300222	148.4985198	B1HB2	Full-length	chr9_107284227_107284516^b^	135.0056956	108.3285612	102.4563633	Same—up
				chr9_107302035_107302114	358.8938006							
				chr9_107306379_107306647	179.2885343							
				chr9_107318752_107318879	199.0418759							
				chr9_107321983_107322118	194.6844742							
				chr9_107323890_107324017	157.6747147							
				chr9_107324834_107325004	166.7800603							
RARA	Chromatin remodeling cofactor	No	Full-length	chr17_40348316_40348464	264.966321	H1BH2	Full-length	chr17_40352566_40352785^a^	16.33385634	26.58279889	13.14358956	Up—same
RNF168	Writer	No	Full-length	chr3_196475231_196475312	48.87929838	B1HB2	Full-length	chr3_196499399_196499448^a^	15.55914629	1.041898901	5.470952467	Down—same
				chr3_196483770_196483891	241.4196715							
				chr3_196487399_196487578	559.0102154							
				chr3_196488607_196488683	281.9957217							
RUVBL2	Chromatin remodeling cofactor	No	Fail	chr19_49007302_49007368	59.04042657	B1HB2	Full-length (≥ 1 < 35 samples)	chr19_49014484_49014603^b^	47.65755052	13.18043333	33.28447611	Same—up
				chr19_49009776_49009882	35.42102967			chr19_49011192_49011310^b^	53.64749641	15.75904163	35.37293219	
SET	Chromatin remodeling cofactor	No	Full-length (≥ 1 < 7 samples)	chr9_128691170_128691227	90.43894815	H1BH2	Full-length (≥ 1 < 55 samples)	chr9_128691837_128691856^a^	156.10463	270.2214865	292.7547895	Up—same
				chr9_128691858_128692000^d^	72.31913227			chr9_128691858_128692000^a^^, d^	42.4648848	65.60493112	83.79094344	
				chr9_128693896_128694042	151.4424237							
SF1	RNA interactor	No	Full-length (≥ 1 < 7 samples)	chr11_64764606_64766155	26.12431153	B1HB2	Full-length (≥ 1 < 35 samples)	chr11_64769426_64769669^a^	172.4290602	119.3861192	127.4741581	Down—up
				chr11_64768106_64768286	481.6714801							
				chr11_64769022_64769129	287.9247696							
				chr11_64769223_64769338^d^	347.3117268			chr11_64769223_64769338^a^^, d^	268.8584151	177.0664881	189.0964138	
				chr11_64769426_64769609	282.135809							
				chr11_64769964_64770053^d^	189.6875959							
				chr11_64770256_64770408	285.8362465			chr11_64769964_64770053^b^^, d^	263.9851006	166.8656433	179.5980223	
				chr11_64773430_64773505	81.68394169							
				chr11_64776498_64776626	547.7503902							
**SMARCC1**	**Chromatin remodeling cofactor**	**No**	**Full-length**	**chr3_47622207_47622341**^d^	**96.91272122**	**B1HB2**	**Full-length**	**chr3_47622207_47622341**^a^^, d^	**69.77555281**	**82.07564582**	**58.44521961**	**Up—same**^c^
				**chr3_47635190_47635344**^d^	**101.6629144**			**chr3_47765620_47765659**^a^	**109.4318057**	**60.44008143**	**77.52801638**	**Down—same**^c^
				**chr3_47636022_47636136**	**211.0242123**							
				**chr3_47693241_47693300**	**161.5219186**			**chr3_47635190_47635344**^b^^, d^	**68.84826131**	**67.34912737**	**52.97551238**	**Same—up**^c^
				**chr3_47701278_47701402**	**293.2391371**							
				**chr3_47706409_47706530**	**354.2425594**			**chr3_47714415_47714490**^b^	**79.49924111**	**65.37542844**	**51.74915032**	**Same—down**^c^
				**chr3_47710683_47710808**	**239.6062289**	**H1BH2**	**Full-length**	**chr3_47638725_47638780**^a^	**164.273275**	**199.1667454**	**152.0656841**	**Up—same**
				**chr3_47729025_47729094**	**108.5173138**							
				**chr3_47736034_47736126**	**318.1530337**							
				**chr3_47738029_47738110**	**551.5659307**							
				**chr3_47745908_47745993**	**250.9244825**							
				**chr3_47772817_47772936**	**90.04905398**							
TLE4	Chromatin remodeling cofactor	No	Full-length	chr9_79573689_79573786	81.6334674	B1HB2	Full-length (≥ 1 < 35 samples)	chr9_79592157_79592236^b^	467.4843202	474.343387	262.241103	Same—down
				chr9_79652593_79652794	123.9598518							
				chr9_79704783_79704902	79.65856586							
				chr9_79705889_79705942	308.2412894							
				chr9_79706747_79706899	197.0445962							
				chr9_79708118_79708250	82.17201832							
				chr9_79722959_79723035	41.67603336							
TRIM28	Reader	No	Full-length (≥ 1 < 7 samples)	chr19_58547792_58547906	62.21120415	B1HB2	Full-length (≥ 1 < 35 samples)	chr19_58547135_58547511^a^	17.9583947	11.84868698	12.65668525	Down—same
TRMT5	Writer	No	Full-length	chr14_60975475_60976126^d^	112.849635	B1HB2	Full-length	chr14_60975475_60976126^a^^, d^	162.1052502	126.0231338	104.0717295	Down—same
				chr14_60977514_60977638	181.1539029							
				chr14_60979231_60979886	238.7932734							
YBX1	RNA interactor	No	Full-length (≥ 1 < 7 samples)	chr1_42683403_42683466	112.849635	H1BH2	Full-length (≥ 1 < 55 samples)	chr1_42693490_42693523^a^	15.04921986	16.15993097	18.42589961	Up—same
YWHAZ	Reader	No	Full-length	chr8_100916525_100920752	18.15976034	B1HB2	Full-length	chr8_100952850_100953057^a^	7.347282187	9.551674351	6.886102163	Up—same
				chr8_100924135_100924298	110.0538877							
				chr8_100924916_100925039	162.8023477							
				chr8_100951929_100952129	82.9444817							
ZNF541	Chromatin remodeling cofactor	No	Fail	chr19_47521479_47521654	7.42074551	H_1_BH_2_	Fail	chr19_47521854_47521994^a^	20.03276583	10.73806747	14.37279423	Down—same

A fivefold paternally enriched and B) twofold paternally enriched. Visual integrity was based on the UCSC Genome Browser. Mean RE abundance is in Reads per Kilobase per Million (RPKM)

*B*_*1*_*HB*_*2*_ background DBP study arm baseline to crossover to crossback, *H*_*1*_*BH*_*2*_ high-DBP study arm baseline to crossover to crossback

Bold text indicates the RNA Element (RE)-containing RNA (RE-RNA) is significantly altered within both DBP study arms

Italicized text indicates the same RE was found in the baseline to crossover and crossover to crossback comparison

^a^indicates the RE is from the baseline to crossover comparison

^b^indicates the RE is from the crossover to crossback comparison

^c^indicates a pattern of a gene in response to DBP is unknown

^d^indicates the RE is shared between at least one DBP responsive RE and the paternally provided set

^e^indicates the RE is shared between both DBP study arms

**Fig. 3:**
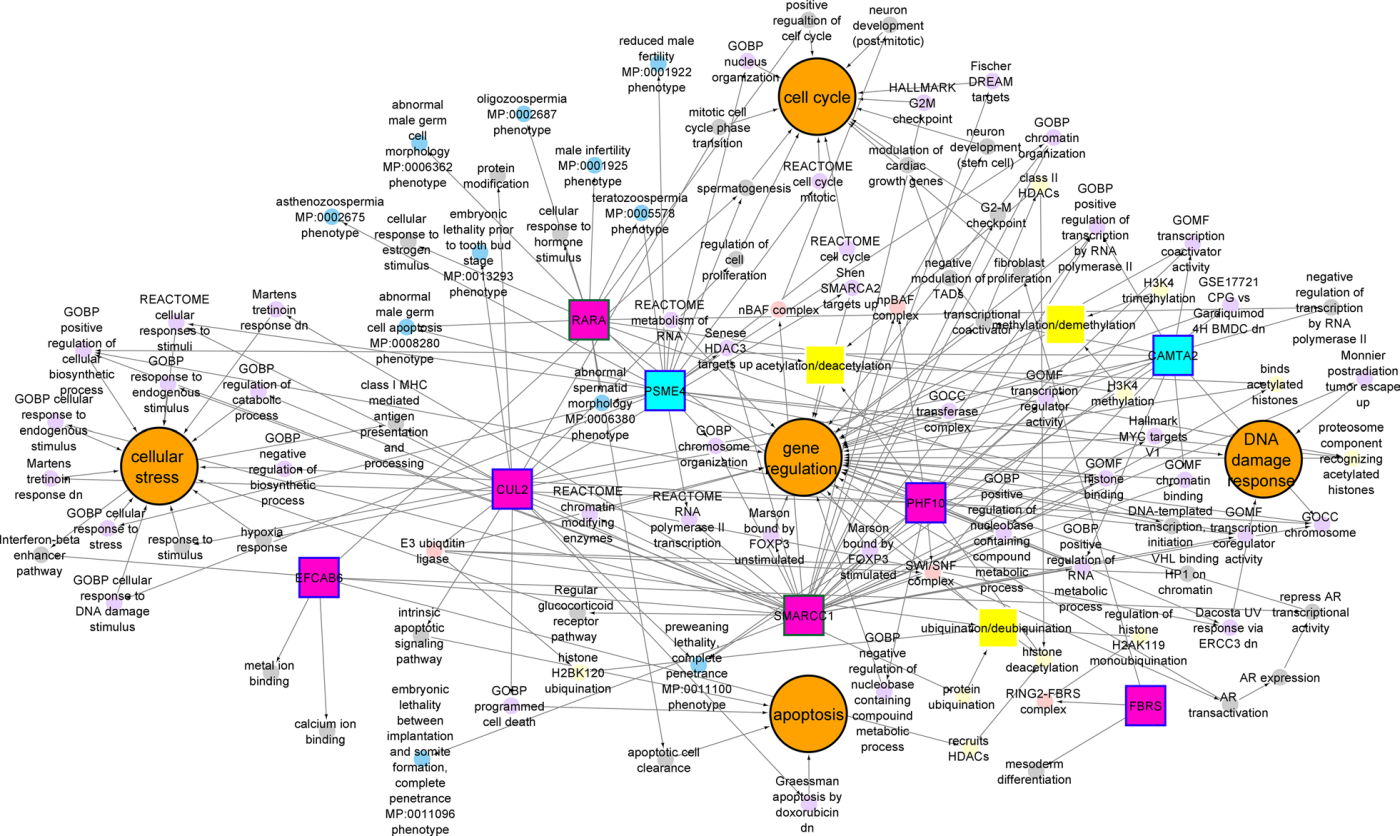
Interconnected gene network of paternally provided dibutyl-phthalate (DBP) responsive RNA Elements (RE)s. The gene network highlights those relationships corresponding to the full-length RE-containing RNAs (RE-RNAs) *CAMTA2, EFCAB6, PSME4, SMARCC1, PHF10*, *RARA*, *FBRS* and *CUL2*. Paternally provided RE-RNA is either fivefold or twofold paternally enriched. Key genes are highlighted based on node color. *Dark blue node borders* indicate fivefold paternal enrichment, while a *green border* indicates twofold paternal enrichment. Node color and shape are as follows; *pink squares*: CRREW (Chromatin remodeler cofactor, RNA interactor, Reader, Eraser, and Writer) RNA, *blue square*: key RNA that is not a CRREW, *orange circle*: major enriched biological pathways, *bright yellow circle*: indicate functions related to acetylation/deacetylation, methylation/demethylation and ubiquination/deubiquination: *light yellow circle*; specific process related to acetylation/deacetylation, methylation/demethylation and ubiquination/deubiquination, *light pink circle*: protein complexes that include at least one key gene, *purple circle*; enriched MSigDB geneset, *grey circle*; gene function not assigned by an MSigDB geneset, *blue circle*; MGI phenotype

As summarized in Table 2, the paternally enriched DBP-responsive RE-RNAs, including *ACSM3*, *PSME4*, *CCDC7*, *NUP98* and *CAMTA2,* responded similarly throughout the study, increasing or decreasing in abundance when exposed to or withdrawn from high-DBP. *CAMTA2* and *PSME4* were full-length (Additional file 8: Table S7), suggesting post-fertilization activity. The corresponding MSigDB gene sets for the aforementioned DBP responsive and paternally provided CRREWs, along with the non-CRREW RE-RNAs *CAMTA2* and *PSME4*, are highlighted in Fig. 3 and Table 3.

**Table 2:** Dibutyl-phthalate (DBP) responsive and paternally provided RNA Element (RE)-containing RNAs (RE-RNAs)

Five fold Paternally enriched				Two fold Paternally enriched			
H_1_B vs B_1_H	H_1_B vs HB_2_	BH_2_ vs B_1_H	BH_2_ vs HB_2_	H_1_B vs B_1_H	H_1_B vs HB_2_	BH_2_ vs B_1_H	BH_2_ vs HB_2_
**ACSM3**	STK39	ANKRD36B	VTI1B	ATXN7	ATXN7	EYA3	PKM
ANKRD36C	ERC1	ANKRD36C	AKT3	SMARCC1	PHC3		
**CAMTA2**	**PSME4**	CTNS	ANKRD36B		SMARCC1		
**CCDC7**	STRN3	CUL2	ERC1				
CTNS		KIAA0586	KIFAP3				
CUL2		MORC2	**NUP98**				
LMBR1L		NPIPA8	PARP6				
MORC2		NUP214	PER1				
NUP214		PER1	PHF10				
**PSME4**		PHF10	**PSME4**				
SEC31A		POLDIP2	RGPD6				
STK39		**PSME4**	SLC22A23				
STRN3		RGPD6	STK39				
		RGPD8					
		STK39					
		ULK4					

RE-RNAs in bold indicate pattern in the same direction upon addition and subtraction of DBP. Paternally provided; five fold paternally enriched or two fold paternally enriched, H_1_B; high-DBP (baseline visit) to background DBP (crossover visit), BH_2_ background DBP (crossover visit) to high-DBP (crossback visit), B_1_H; background DBP (baseline visit) to high-DBP (crossover visit), HB_2_ high-DBP (crossover visit) to background DBP (crossback visit).  Bold text indicates the RE-RNA is shared within both drug study arms.

**Table 3:** Enriched biological processes of key dibutyl-phthalate (DBP) responsive, paternally provided RNA Element (RE)-containing RNAs (RE-RNAs)

RE-RNA	CRREW	CRREW class	Enriched biological process	Enriched MSigDB Gene Set
CAMTA2	No	–	Cellular stress	GOBP positive regulation of cellular biosynthetic process
			Gene regulation	GOBP positive regulation of nucleobase containing compound metabolic process
				GOBP positive regulation of RNA metabolic process
				GOBP positive regulation of transcription by RNA polymerase II
				GOCC chromosome
				GOMF chromatin binding
				GOMF transcription coactivator activity
				GOMF transcription regulator activity
CUL2	Yes	Chromatin remodeler cofactor	Cellular stress	REACTOME cellular responses to stimuli
			Apoptosis	Graessman apoptosis by doxorubicin dn
				GOBP programmed cell death
			DNA damage response to UV exposure	Dacosta UV response via ERCC3 dn
			Gene regulation	GOCC transferase complex
				Shen SMARCA2 targets up
PHF10	Yes	Chromatin remodeler cofactor	Cellular stress	GOBP negative regulation of biosynthetic process
				GOBP positive regulation of cellular biosynthetic process
				Martens tretinoin response dn
			Gene regulation	GOBP negative regulation of nucleobase containing compound metabolic process
				GOBP positive regulation of nucleobase containing compound metabolic process
				GOBP positive regulation of RNA metabolic process
				GOBP positive regulation of transcription by RNA polymerase II
				GOCC chromosome
				GOMF histone binding
				GOMF transcription coregulator activity
				GOMF transcription regulator activity
PSME4	No	–	Cellular stress	GOBP cellular response to DNA damage stimulus
				GOBP cellular response to stress
				REACTOME cellular responses to stimuli
			Cell cycle	GOBP nucleus organization
				REACTOME cell cycle
				REACTOME cell cycle mitotic
			DNA damage response to UV exposure	Dacosta UV response via ERCC3 dn
			Gene regulation	GOBP chromatin organization
				GOBP chromosome organization
				GOMF histone binding
				REACTOME metabolism of RNA
				REACTOME RNA polymerase II transcription
				Senese HDAC3 targets up
SMARCC1	Yes	Chromatin remodeler cofactor	Cellular stress	GOBP cellular response to endogenous stimulus
				GOBP positive regulation of cellular biosynthetic process
				GOBP regulation of catabolic process
				GOBP response to endogenous stimulus
				Martens tretinoin response dn
			Cell cycle	Fischer DREAM targets
				Hallmark G2M checkpoint
			DNA damage response to UV exposure	Monnier postradiation tumor escape up
			Gene regulation	GOBP chromatin organization
				GOBP chromatin binding
				GOBP chromosome organization
				GOBP positive regulation of RNA metabolic process
				GOBP positive regulation of nucleobase containing compound metabolic process
				GOBP positive regulation of transcription by RNA polymerase II
				GOCC chromosome
				GOMF histone binding
				GOMF transcription coactivator activity
				GOMF transcription coregulator activity
				GOMF transcription regulator activity
				GSE17721 CPG vs gardiquimod 4 h BMDC dn
				Hallmark MYC targets V1
				Marson bound by FOXP3 stimulated
				Marson bound by FOXP3 unstimulated
				REACTOME chromatin modifying enzymes
				REACTOME RNA polymerase II transcription
				Senese HDAC3 targets up

RE-RNAs are visually full-length using the UCSC genome browser. DNA damage response: DNA damage response to UV exposure. Paternally provided: fivefold paternally enriched or twofold paternally enriched; CRREW: chromatin remodeler cofactor, RNA interactor, reader, eraser and writer.

A total of 46 of the 132 DBP responsive and paternally provided RE-RNAs were not within any of the enriched MSigDB gene sets related to the biological processes highlighted in Fig. 2. These 46 RE-RNAs were evaluated separately using EnrichR to identify gene function (Additional file 9: Table S8) and determine whether they reported functions within these biological processes. This highlighted multiple full-length CRREWs. First is the histone deacetylase complex (HDAC) [27, 28] regulated *EFCAB6*, a chromatin remodeler cofactor associated with androgen receptor (AR) signaling, proteolysis, and transcriptional regulation. It further highlighted the chromatin remodeler cofactor *RARA,* which functions in histone methylation [29, 30], is involved in apoptotic cell clearance, the positive regulation of the cell cycle, cellular response to estrogen and hormone stimulus, and the negative regulation of transcription by RNA polymerase II (Additional file 9: Table S8). Interestingly, the chromatin remodeler cofactor *FBRS* had no reported ontologies within EnrichR. However, it is part of the RING2-FBRS replication-independent complex involved in histone modification [29, 31, 32].

## Discussion

Utilizing the DBP-responsive sperm RE-RNAs [5], fivefold paternally enriched RE-RNAs [3], and twofold paternally enriched RE-RNAs, we have begun to frame the effect of exposures on what is paternally provided at fertilization. Significantly more than expected RE-RNAs corresponding to CRREWs (38 DBP responsive, paternally provided CRREWs within shared enriched MSigDB gene sets) were identified. This included three full-length RE-RNAs, consistent with the view that they act as mediators between pathways in response to exposure. This increase in the proportion of CRREWs but not TFs mimics what we previously reported within a series of body mass index (BMI) responsive RE-RNAs [4]. Consistent with the above, if they play a role during protamine replacement and syngamy as the blastocyst begins to form, one would expect a more significant proportion of CRREW RE-RNAs than TFs remaining in the mature sperm.

DBP impacts 86 paternally provided RE-RNAs (Figs. 2B, 3) ontologically linked to cellular stress, cell cycle, apoptosis, DNA damage response, and gene regulation. While most of these RE-RNAs appear responsive to DBP in either the H_1_BH_2_ or B_1_HB_2_ exposure comparisons, a subset is responsive irrespective of exposure duration or time removed from high-DBP. Three full-length CRREWs (*CUL2*, *PHF10,* and *SMARRC1*) and two full-length fivefold paternally enriched non-CRREW RE-RNAs (*CAMTA2* and *PSME4*) were identified. These five RE-RNAs respond to the addition or removal of high-DBP irrespective of the study arm and are within a series of biological processes, as represented in Fig. 3.

This analysis identified a highly complex, interconnected gene network (Fig. 1D) reflecting DBP responsive and paternally provided RE-RNAs. To more readily interpret this gene network, the focus was given to the full-length DBP responsive paternally provided RE-RNAs highlighted in Fig. 3. Here, *SMARCC1* and *PHF10* (top of Fig. 3) function as part of the SWI/SNF complex (middle left of Fig. 3) to enhance the transactivation of AR [33–35] in direct opposition to the AR transcriptional repression by the chromatin remodeling cofactor *EFCAB6* (Additional file 9: Table S8, bottom of Fig. 3) [27, 28]. Interestingly, DBP has anti-androgenic activity (reviewed in [13]); however, AR was not found as directly responsive to high-DBP exposure or withdrawal [5], which is consistent with the lack of an in vitro interaction between DBP and AR [36]. In response to DNA damage, the *SMARCC1* and *PHF10* containing SWI/SNF complex (middle left of Fig. 3) are known to accumulate, likely enabling transient chromatin accessibility to DNA-binding and DNA damage response proteins [37]. As expected, *PHF10* and *SMARCC1* were within a number of the same enriched gene sets (Table 3, Fig. 3), including *GOMF histone binding* alongside *PSME4*, and *GOMF transcription regulator activity* and *GOBP positive regulation of transcription by RNA polymerase II* with *CAMTA2*, indicating shared functions*. CAMTA2*, while not a CRREW, is a transcriptional activator that associates with class II HDACs to negatively modulate topological associated domains (TADs) [38, 39]. However, the proteasome component PSME4 acts to recognize acetylated histones, promoting histone degradation during spermatogenesis and the DNA damage response [40, 41]. The chromatin remodeler cofactor *RARA* regulating transcription in a ligand dependent manner (Additional file 9: Table S8). *RARA* functions in response to estrogen stimulus (Additional file 9: Table S8), is involved in H3K4 methylation [30] as part of a heterodimer and induces histone deacetylation when the heterodimer associates with specific multiprotein complexes [42]. These relationships begin to highlight how the DNA damage response may feedforward regulating gene expression (center of Fig. 3). Together with *PSME4*, *CUL2* was within the enriched DNA damage response gene set *Dacosta UV response *via* ERCC3 dn* (Table 3, Fig. 3). As part of the E3 ubiquitin ligase complex (middle left of Fig. 3), CUL2 alongside BAF250, elongin C and ROC1 ubiquitinate histone H2BK120 aiding in SWI/SNF complex H3K4 trimethylation [43, 44]. CUL2 further enables the interaction of VHL with elongin B and elongin C to form the E3 ubiquitin ligase complex to recruit VHL to HP1 chromatin [45, 46]. In addition, the CUL2 containing E3 ubiquitin ligase has been identified as important in Adenovirus inactivation of a DNA damage response [47]. It is integral to the progression of G1 to S and the S-phase-dependent DNA damage response [48]. While not identified within the enriched MSigDB gene sets from Additional file 3: Table S2, *RARA* participates in the regulation of the cell cycle and apoptotic cell clearance (Additional file 9: Table S8, bottom of Fig. 3). This indicates a potential interaction with CUL2 within the apoptotic gene sets *Graessman apoptosis by doxorubicin dn* and *GOBP programmed cell death* (Table 3, Fig. 3). These relationships highlight the potential cooperation between these CRREW complexes and how a DBP-induced DNA damage response may impact the cell cycle, leading to its arrest and eventually, cell death.

*CUL2* and *PSME4* were also within *REACTOME cellular responses to stimuli* (Table 3, Fig. 3). While little is known about the function of FBRS, it is part of the RING2-FBRS complex (bottom right of Fig. 3), a type of Polycomb group (PcG) complex [29, 31, 32] that acts as a transcriptional activator of mesoderm differentiation, and a regulator of H2AK119ub1 levels [49]. *PSME4* and *SMARCC1* are within the gene sets *REACTOME RNA polymerase II transcription*, *Senese HDAC3 targets up,* and *GOBP chromosome organization* (Table 3, Fig. 3). With DBP-responsive RE-RNAs represented within each enriched biological process, an interconnected network centered on cellular response to DNA damage as modulated by CRREWs was highlighted (Figs. 2A, 3). This is consistent with a known effect of phthalate exposure resulting in DNA damage [50, 51].

Intriguingly, the fivefold paternally enriched *CUL2*, *PHF10*, *EFCAB6,* and *FBRS,* and twofold paternally enriched *SMARCC1* and *RARA* are chromatin remodeler cofactors (Fig. 3, Additional file 5: Table S4) providing a foray into mechanism. As shown in Fig. 3, the majority of these eight DBP responsive and paternally provided RE-RNAs are involved in acetylation or deacetylation [27, 28, 38, 39, 44, 46, 52], except *CUL2* and *FBRS* (Fig. 3). This highlights the importance of paternally derived acetylation factors during the final steps of spermatogenesis and, potentially, early embryogenesis. During spermatogenesis, acetylation of histone H4 is a critical step in replacing histones with protamine (reviewed in [^53^, ^54^]). Within 3 h of fertilization, the paternal chromatin will undergo transient hyperacetylation of histone H4 (reviewed in [^53^, ^55^]). To date, the molecular components integral to transient hyperacetylation remain elusive [53]. These RNAs may function in this transient hyperacetylation event.

## Conclusions

Alterations in mouse sperm RNAs have been linked to offspring's metabolic health and stress response [2, 6, 8–11]. These studies have provided evidence in favor of the paternal origins of health and disease (POHaD) (reviewed in [12, 56, 57]). Recently, environmental exposures, including DBP and bisphenol A (BPA), and lifestyle factors such as BMI have been associated with alterations of epigenetic marks in sperm [4, 5, 12, 58–61], that is beginning to reconcile exposure and POHaD. Each of the CRREWs highlighted (*CUL2*, *SMARCC1*, *PHF10*, *EFCAB6*, *FBRS*, and *RARA*) alongside the non-CRREW *CAMTA2* and *PSME4* are paternally delivered as full-length RNAs ready for translation and early utilization in the fertilized oocyte. Perhaps these three CRREWs play a role directly following fertilization as the father’s chromatin is restructured or during syngamy. Interestingly, these genes are not represented within the human oocyte proteome [62], although *CUL2*, *PSME4,* and *EFCAB6* are within the human sperm proteome [63]. As described above, they may encode early transient events like hyperacetylation in response to DBP exposure. On one hand, these RNAs are likely essential in functions prior to Embryonic Genome Activation, consistent with the MGI phenotype Ontology Annotations [64]. For example, *CAMTA2*, *CUL2*, *PHF10,* and *SMARCC1* mouse knockdowns result in embryonic and/or preweaning lethality (Fig. 3, blue circles). On the other hand, *PSME4* and *RARA* knockdowns impair male fertility due to several abnormalities related to spermatogenesis [64] (Fig. 3, blue circles). This emphasizes the importance of the sperm providing full-length transcripts and proteins at fertilization, as they may serve as the driving force behind the DBP-induced decreases in semen and embryo quality and subsequent increases in time to pregnancy.

## Methods

### DBP responsive REs

The differentially expressed REs summarized in Estill, MS et al. [5] from the crossover–crossback designed study were utilized (Additional file 2: Table S1). Men entered the study were on either a high-DBP-coated mesalamine at baseline (high-DBP study arm ( +), 112 semen samples, Fig. 1A) or non-DBP-coated mesalamine at baseline (background DBP study arm (−), 63 semen samples, Fig. 1A) [5]. It is important to note that both medications contain the same active pharmaceutical, mesalamine, and were exchangeably prescribed to IBD patients. They differed only in the presence of DBP in the coating. The 90-day intervals were designed to be reflective of a spermatogenic cycle and hence washout, when men would switch to the opposing drug from baseline to crossover, B_1_H (background DBP to high-DBP)/H_1_B (high-DBP to background DBP), then switched back from crossover to crossback, HB_2_ (high-DBP to background DBP)/BH_2_ (background DBP to high-DBP). Here, REs were evaluated as a function of this 90-day spermatogenic cycle and the duration of high-DBP exposure/withdrawal. This study was approved by the institutional review boards Partners Hospitals (Massachusetts General Hospital) protocol 2005P001631 and of Harvard T.H. Chan School of Public Health, Beth Israel Deaconess Medical Center, and Brigham and Women’s Hospital. The use of human tissue was approved by the Wayne State University Investigation Committee and carried out under the Wayne State University Human Investigation Committee IRB protocol 095701MP2E(5R).

### Paternally provided REs

Paternally provided REs (generated from 7 non-IBD semen samples not exposed to high-DBP) were characterized from a total of 75,988 REs [11,386 RE–RE-containing RNAs (RE-RNAs)] identified within the zygote having a reads per kilobase per million (RPKM) > 10 [3]. As the zygote has yet to undergo embryonic genome activation, RE-RNAs present will be those provided directly by the sperm and oocyte [3]. Paternally enriched REs delivered at fertilization in which enrichment was at least fivefold higher when compared to the oocyte (fivefold paternally enriched) were described as having a median abundance > 25 RPKM in sperm, < 5 RPKM in the oocyte, and > 10 RPKM in the zygote [3]. This yielded a series of stringent REs that the father provides at fertilization.

To expand upon what may be paternally provided, an additional set of twofold paternally enriched REs were defined using a lower enrichment threshold for comparison (Fig. 1A, B). From the total 51,089 zygotic REs (10,277 RE-RNAs) independent of the paternally or maternally enriched REs previously defined [3], the paternal RE/maternal RE ratio was calculated in the following manner. If the RPKM of the zygotic RE was larger than the sum of the sperm and oocyte REs, the contribution of the sperm and oocyte equaled their respective RPKM abundance. If the zygote RPKM was less than the sum of the sperm plus oocyte REs, paternal contribution (Pc) was calculated as $Pc = Z - \left( {\frac{Z}{{1 + \left( {\frac{s}{o}} \right)}}} \right)$, where *Z* represents the zygote REs RPKM, *s* represents the sperm RE RPKM and *o* represents the oocyte RE RPKM. Paternally contributed REs at a level twofold greater than the maternal contribution were termed twofold paternally enriched REs. While a series of these paternally provided REs are enriched in the sperm compared to the oocyte, some are specific to the sperm. For REs specific to the sperm, the RPKM in the oocyte was  < 2, the abundance value in which true RE presence cannot be confirmed. Each set of paternally provided REs was evaluated as a separate and combined RE list, as defined in Fig. 1.

### Sample and transcript integrity

The transcript Integrity Index (TII) algorithm [26] was used to identify samples of similar quality [3, 5] using the 22 stable sperm-specific transcripts we previously defined [26]. The TII threshold was set at 50% of the transcript covered by at least 5 reads per million (RPM). Samples within the fourth quartile (Q4) were considered to have poor quality RNA [26]. Those samples passing TII were used to visually assess the paternally delivered RE corresponding RNAs of interest using the UCSC Genome Browser using Gencode version 36 [65]. RNAs were considered full-length if a minimum of 5 RPKM covered the transcript in all samples (7 paternally provided samples, 55 high-DBP study arm samples, 35 background DBP study arm samples). The 5 RPKM cutoff defines the minimum abundance in which there can be confidence in RE presence.

### Gene ontology and statistical analysis

Enriched biological processes and pathways were evaluated using the Molecular Signature Database (MSigDB) version 7.5.1, employing the following collections: Hallmark gene sets (Hm), curated gene sets (C2), Gene Ontology (GO) gene sets (C5), and immunologic gene sets (C7). The collection C3: regulatory target gene set sub-category transcription factor (TF) targets were used to identify biologically corresponding TFs within the data. Each collection was considered separately. Thresholds were set to return the top 100 gene sets with a False Discovery Rate (FDR) *q* < 0.05 and a minimum of a two-gene overlap.

MSigDB analysis enables a maximum of 500 recognized genes per analysis. To evaluate the DBP responsive REs in Additional file 2: Table S1, each DBP comparison in Fig. 1A was separated into six groups based on the empirical (bootstrapped) *p* value that was generated using random resampling [5]. To group REs into the six empirical *p* value range groups, the REs within the largest DBP responsive comparison (all significantly associated REs within the(B_1_H comparison [5], Fig. 1B) were used. This would ensure that no comparison visualized in Fig. 1B would contain more than 500 unique RE-RNAs for MSigDB investigation. The six empirical *p* value range groups were as follows: group 1 = *p* < 0.013, group 2 = *p* between 0.013 and 0.023, group 3 = *p* between 0.023 and 0.032, group 4 = *p* between 0.032 and 0.041, group 5 = *p* between 0.041 and 0.045, and group 6 = *p* between 0.045 and 0.05. In the B_1_H, all correlated REs within Fig. 1B (3,651 [2,311 genes] REs), this segregated the REs as follows; group 1 = 711 REs (577 unique genes with 485 genes recognized), group 2 = 706 REs (566 unique genes with 486 genes recognized), group 3 = 718 REs (565 unique genes with 477 genes recognized), group 4 = 693 REs (551 unique genes with 470 genes recognized), group 5 = 451 REs (374 unique genes with 323 genes recognized), and group 6 = 372 REs (311 unique genes with 250 genes recognized).

EnrichR [66, 67], along with GeneCards (https://www.genecards.org/) [68] and the NIH Genetics Home Reference (https://ghr.nlm.nih.gov/), were utilized to assess gene function and disease associations. EnrichR categories of *Pathways*, *Ontologies,* and *Diseases/Drugs* were considered. Mediators of gene expression above TFs, considered Chromatin remodeler cofactors, RNA interactors, Readers, Erasers, and Writers (CRREWs). Briefly, CRREWs were identified from the curated list as described [4, 63], and key transcripts of interest were evaluated as part of the human sperm proteome.

The significance of proportional overlaps for TFs and CRREWs within the data was determined by the hypergeometric probability test with normal approximation from http://nemates.org/MA/progs/overlap_stats.html. This provides a *p* value corresponding to a representation factor value indicating whether the overlap is significantly more or less than expected. A two-tailed *t* test for two samples of unequal variance was performed to calculate *p* values associated with the paternal/maternal contribution fold change using Microsoft 365 Excel (version 2202).

## Supplementary Information

**Additional file 1: Figure S1. **Chromatin remodeler cofactor, RNA interactor, reader, eraser and writer (CRREW) RNA Element (RE)-containing RNA (RE-RNA) visual integrity. Representative samples chosen for A) *CUL2*, B) *SMARCC1* and C) *PHF10*. Integrity of DBP responsive and paternally provided CRREWs was determined using the UCSC Genome Browser Gencode version 41 track. Threshold for an RE-RNA to be considered full-length was set at a minimum of 5 Reads per Kilobase per Million (RPKM) across all transcript exons in all 7 paternally provided samples and all DBP responsive samples (high-DBP study arm (H_1_BH_2_), 55 samples; background-DBP study (B_1_HB_2_) arm: 35 samples).

**Additional file 2: Table S1. **Number of RNA elements (REs) responsive to dibutyl-phthalate (DBP). REs were obtained from the publication Estill, MS et al. (2019b) (5). H_1_B; high-DBP (baseline visit) to background DBP (crossover visit), BH_2_; background DBP (crossover visit) to high-DBP (crossback visit), B_1_H; background DBP (baseline visit) to high-DBP (crossover visit), HB_2_; high-DBP (crossover visit) to background DBP (crossback visit).

**Additional file 3: Table S2. **Enriched biological processes and pathways related to cellular stress, cell cycle, apoptosis, DNA damage response and gene regulation. RNA Element (RE) indicated in Fig. 1 panel B processes were used to query the Molecular Signature Database. Bolded text indicates the gene set is enriched in the paternally provided and DBP responsive RE-containing RNAs (RE-RNAs) evaluated. Italicized text indicates a gene set enriched upon DBP exposure addition and withdraw irrespective of original study arm. H_1_B; high-DBP (baseline visit) to background DBP (crossover visit), BH_2_; background DBP (crossover visit) to high-DBP (crossback visit), B_1_H; background DBP (baseline visit) to high-DBP (crossover visit), HB_2_; high-DBP (crossover visit) to background DBP (crossback visit), purple text; gene sets related to cellular stress, brown text; gene sets related to the cell cycle, green text; gene sets related to apoptosis and cell death, red text; gene sets related to DNA damage response (DNA damage response to UV exposure), blue text; gene sets related to gene regulation., indicates no enrichment in the gene set.

**Additional file 4: Table S3.** Number of dibutyl-phthalate (DBP) responsive transcription factor (TF) binding site gene sets and Chromatin remodeler cofactors, RNA interactors, Readers, Erasers and Writers (CRREWs). A) Enriched TF binding site gene sets. Enriched gene sets were separated based on the identification of having a known TF reported to bind. B) Number of DBP responsive CRREWs. Gene lists were generated from those RNA Element (RE)-containing RNAs (RE-RNAs) represented within Fig. 1. Representation factor and *p* value were determined by hypergeometric probability test.

**Additional file 5: Table S4. **All Di-butyl phthalate (DBP) responsive and paternally provided CRREWs within sperm. H_1_B; high-DBP (baseline visit) to background DBP (crossover visit), BH_2_; background DBP (crossover visit) to high-DBP (crossback visit), B_1_H; background DBP (baseline visit) to high-DBP (crossover visit), HB_2_; high-DBP (crossover visit) to background DBP (crossback visit), paternally provided, RE-containing RNAs (RE-RNAs) that are fivefold paternally enriched or twofold paternally enriched.

**Additional file 6: Table S5. **REs unique to the zygote that are twofold paternally enriched. A) DBP responsive and 2x paternally enriched. B) 2x paternally enriched but not DBP responsive. REs required the paternal contribution to be > twofold the maternal, or the maternal RE abundance to be < 2 RPKM and paternal RE abundance < 25 and > 2 RPKM. #DIV/0 indicates contribution is solely from the father. Green fill indicates the RE is shared between two genes in the sperm contributed set.

**Additional file 7: Table S6. **Number of paternally provided Chromatin remodeler cofactors, RNA interactors, readers, erasers and writers (CRREWs). A) Number of paternally provided CRREWs. B) Number of dibutyl-phthalate (DBP) responsive, paternally provided CRREWs. Representation factor and *p* value were determined by hypergeometric probability test. Paternally provided CRREWs include those that are fivefold paternally enriched and twofold paternally enriched.

**Additional file 8: Table S7. **Integrity of specifically paternally provided and di-butyl phthalate (DBP) responsive transcripts. RNA Element (RE)-containing RNAs (RE-RNAs) represented respond to DBP addition and subtraction in the same direction. Visual inspection is based on the UCSC Genome Browser. Bold text indicates the RE is shared between the paternally provided and DBP responsive samples, blue text indicates the DBP responsive RE was associated with the baseline to crossover (B_1_H or H_1_B) comparison and red text indicates the DBP responsive RE was associated with the crossover to crossback comparison (HB_2_ or BH_2_). Paternally provided indicates samples in which the fivefold paternally enriched and twofold paternally enriched REs were obtained. B_1_HB_2_; background DBP study arm baseline to crossover to crossback, H_1_BH_2_; high-DBP study arm baseline to crossover to crossback. Mean RE abundance is in Reads per Kilobase per Million (RPKM).

**Additional file 9: Table S8. **Gene Ontology of dibutyl-phthalate (DBP) responsive, paternally provided RNA Element (RE)-containing RNAs (RE-RNAs) not within the enriched biological processes. Ontology was assigned using EnrichR. Bold text; indicates function related to the enriched biological processes highlighted in Fig. 2B, italics; indicate an ontology that is shared by at least two RE-RNAs, na; no ontology reported, not within EnrichR; the RE-RNA is not recognized by EnrichR, …; no relevance to enriched biological processes highlighted in Fig. 2B, B_1_HB_2_; background DBP study arm baseline to crossover to crossback, H_1_BH_2_; high-DBP study arm baseline to crossover to crossback, Paternally provided; fivefold paternally enriched and twofold paternally enriched RE-RNAs.

## Data Availability

Sequencing data for the DBP responsive REs are deposited as a GEO data set with the following accession number: GSE129216 and referenced in [5]. The REs utilized to determine the 5 × enriched and 2 × enriched paternally provided REs defined in [3]. Oocyte and zygote sequences were downloaded from the GEO database accessions GSE44183 and GSE71318.

## Abbreviations

AR: Androgen receptor
B: Men transitioning from the high-DBP mesalamine to background DBP mesalamine (crossover visit)
B_1_: Men entering on the non-DBP mesalamine (background DBP baseline visit)
B_1_H: Background DBP (baseline visit) to high-DBP (crossover visit)
B_2_: Men returning to non-DBP mesalamine (background DBP crossback visit)
BH_2_: Background DBP (crossover visit) to high-DBP (crossback visit)
B_1_HB_2_: Background DBP study arm, baseline to crossover to crossback
BMI: Body mass index
BPA: Bisphenol A
C2: Curated gene sets
C3: Regulatory target gene set sub-category transcription factor targets
C5: GO gene sets
C7: Immunologic gene sets
CRREWs: Chromatin remodeler cofactors, RNA interactors, Readers, Erasers, and Writers
DBP: Dibutyl-phthalate
EPA: Environmental protection agency
FDR: False discovery rate
GO: Gene ontology
H: Men transitioning from the non-DBP mesalamine to high-DBP mesalamine (crossover visit)
H_1_: Men entering on the high-DBP mesalamine (high-DBP baseline visit)
H_1_B: High-DBP (baseline visit) to background DBP (crossover visit)
H_1_BH_2_: High-DBP study arm, baseline to crossover to crossback
H_2_: Men returning to high-DBP mesalamine (high-DBP crossback visit)
HB_2_: High-DBP (crossover visit) to background DBP (crossback visit)
HDACs: Histone deacetylase complexes
Hm: Hallmark gene sets
IBD: Irritable bowel disease
MBP: Monobutyl phthalate
MSigDB: Molecular signatures database
nBAF complex: Neuron-specific chromatin remodeling complex
NHANES: National Health and Nutrition Examination Survey
npBAF complex: Neural progenitors-specific chromatin remodeling complex
RE: RNA Element
Paternally provided REs: 5-Fold paternally enriched/twofold paternally enriched REs
PDT: Photodynamic therapy
PcG: Polycomb group
POHaD: Paternal developmental origins of health and diseaseQ4: fourth quartile
REDa: RNA element discovery algorithm
RE: RNA element
RE-RNA: RE-containing RNA/gene
RPKM: Reads per kilobase per million
RPM: Reads per million
TAD: Topological associated domain
TF: Transcription factor
TII: Transcript integrity index

## References

[1] Ostermeier GC, Miller D, Huntriss JD, Diamond MP, Krawetz SA (2004). Delivering spermatozoan RNA to the oocyte. Nature.

[2] Godia M, Swanson G, Krawetz SA (2018). A history of why fathers' RNA matters. Biol Reprod.

[3] Estill MS, Hauser R, Krawetz SA (2019). RNA element discovery from germ cell to blastocyst. Nucleic Acids Res.

[4] Swanson G, Estill M, Diamond MP, Legro RS, Coutifaris C, Barnhart MD (2019). Human Chromatin remodeler cofactor interactor, eraser and writer sperm RNAs responding to obesity. Epigenetics.

[5] Estill M, Hauser R, Nassan FL, Moss A, Krawetz SA (2019). The effects of di-butyl phthalate exposure from medications on human sperm RNA among men. Nat Sci Rep.

[6] Gapp K, Jawaid A, Sarkies P, Bohacek J, Pelczar P, Prados J (2014). Implication of sperm RNAs in transgenerational inheritance of the effects of early trauma in mice. Nat Neurosci.

[7] Donkin I, Versteyhe S, Ingerslev LR, Qian K, Mechta M, Nordkap L (2016). Obesity and bariatric surgery drive epigenetic variation of spermatozoa in humans. Cell Metab.

[8] Grandjean V, Fourré S, De Abreu DAF, Derieppe M-A, Remy J-J, Rassoulzadegan M (2015). RNA-mediated paternal heredity of diet-induced obesity and metabolic disorders. Sci Rep.

[9] de Castro BT, Ingerslev LR, Alm PS, Versteyhe S, Massart J, Rasmussen M (2016). High-fat diet reprograms the epigenome of rat spermatozoa and transgenerationally affects metabolism of the offspring. Mol Metab.

[10] Chen Q, Yan M, Cao Z, Li X, Zhang Y, Shi J (2016). Sperm tsRNAs contribute to intergenerational inheritance of an acquired metabolic disorder. Science.

[11] Carone BR, Fauquier L, Habib N, Shea JM, Hart CE, Li R (2010). Paternally induced transgenerational environmental reprogramming of metabolic gene expression in mammals. Cell.

[12] Marcho C, Oluwayiose OA, Pilsner JR (2020). The preconception environment and sperm epigenetics. Andrology.

[13] Hauser R, Calafat AM (2005). Phthalates and human health. Occup Environ Med.

[14] Lyche JL, Gutleb AC, Bergman Å, Eriksen GS, Murk AJ, Ropstad E (2009). Reproductive and developmental toxicity of phthalates. J Toxicol Environ Health, Part B.

[15] Nassan FL, Coull BA, Gaskins AJ, Williams MA, Skakkebaek NE, Ford JB (2017). Personal care product use in men and urinary concentrations of select phthalate metabolites and parabens: results from the environment and reproductive health (EARTH) study. Environ Health Perspect.

[16] Hauser R, Duty S, Godfrey-Bailey L, Calafat AM (2004). Medications as a source of human exposure to phthalates. Environ Health Perspect.

[17] Nassan FL, Korevaar TIM, Coull BA, Skakkebæk NE, Krawetz SA, Estill M (2019). Dibutyl-phthalate exposure from mesalamine medications and serum thyroid hormones in men. Int J Hyg Environ Health.

[18] Hernández-Díaz S, Mitchell AA, Kelley KE, Calafat AM, Hauser R (2009). Medications as a potential source of exposure to phthalates in the US population. Environ Health Perspect.

[19] Bloom M, Whitcomb B, Chen Z, Ye A, Kannan K, Buck LG (2015). Associations between urinary phthalate concentrations and semen quality parameters in a general population. Hum Reprod.

[20] Wang Y-X, You L, Zeng Q, Sun Y, Huang Y-H, Wang C (2015). Phthalate exposure and human semen quality: Results from an infertility clinic in China. Environ Res.

[21] Wu H, Ashcraft L, Whitcomb BW, Rahil T, Tougias E, Sites CK (2017). Parental contributions to early embryo development: influences of urinary phthalate and phthalate alternatives among couples undergoing IVF treatment. Hum Reprod.

[22] Louis GMB, Sundaram R, Schisterman EF, Sweeney A, Lynch CD, Kim S (2014). Semen quality and time to pregnancy: the longitudinal investigation of fertility and the environment study. Fertil Steril.

[23] Nassan FL, Coull BA, Skakkebaek NE, Williams MA, Dadd R, Mínguez-Alarcón L (2016). A crossover-crossback prospective study of dibutyl-phthalate exposure from mesalamine medications and semen quality in men with inflammatory bowel disease. Environ Int.

[24] Centers for Disease Control and Prevention, Fourth National Report on Human Exposure to Environmental Chemicals: CDC. 2021 http://www.cdc.gov/biomonitoring/pdf/FourthReport_UpdatedTables_Feb2015.pdf. Accessed 20 Aug 2022

[25] Shannon P, Markiel A, Ozier O, Baliga NS, Wang JT, Ramage D (2003). Cytoscape: a software environment for integrated models of biomolecular interaction networks. Genome Res.

[26] Swanson GM, Estill MS, Krawetz SA (2022). The transcript integrity index (TII) provides a standard measure of sperm RNA. Syst Biol Reprod Med.

[27] Niki T, Takahashi-Niki K, Taira T, Iguchi-Ariga SM, Ariga H (2003). DJBP: a novel DJ-1-binding protein, negatively regulates the androgen receptor by recruiting histone deacetylase complex, and DJ-1 antagonizes this inhibition by abrogation of this complex. Mol Cancer Res.

[28] Jin W (2020). Novel insights into PARK7 (DJ-1), a potential anti-cancer therapeutic target, and implications for cancer progression. J Clin Med.

[29] Medvedeva YA, Lennartsson A, Ehsani R, Kulakovskiy IV, Vorontsov IE, Panahandeh P (2015). EpiFactors: a comprehensive database of human epigenetic factors and complexes. Database (Oxford).

[30] Fujiki R, Chikanishi T, Hashiba W, Ito H, Takada I, Roeder RG (2009). GlcNAcylation of a histone methyltransferase in retinoic-acid-induced granulopoiesis. Nature.

[31] Schwartz YB, Pirrotta V (2013). A new world of Polycombs: unexpected partnerships and emerging functions. Nat Rev Genet.

[32] Gao Z, Zhang J, Bonasio R, Strino F, Sawai A, Parisi F (2012). PCGF homologs, CBX proteins, and RYBP define functionally distinct PRC1 family complexes. Mol Cell.

[33] Banga SS, Peng L, Dasgupta T, Palejwala V, Ozer HL (2009). PHF10 is required for cell proliferation in normal and SV40-immortalized human fibroblast cells. Cytogenet Genome Res.

[34] Staahl BT, Crabtree GR (2013). Creating a neural specific chromatin landscape by npBAF and nBAF complexes. Curr Opin Neurobiol.

[35] Phelan ML, Sif S, Narlikar GJ, Kingston RE (1999). Reconstitution of a core chromatin remodeling complex from SWI/SNF subunits. Mol Cell.

[36] Foster PM, Mylchreest E, Gaido KW, Sar M (2001). Effects of phthalate esters on the developing reproductive tract of male rats. Hum Reprod Update.

[37] Izhar L, Adamson B, Ciccia A, Lewis J, Pontano-Vaites L, Leng Y (2015). A systematic analysis of factors localized to damaged chromatin reveals PARP-dependent recruitment of transcription factors. Cell Rep.

[38] Song K, Backs J, McAnally J, Qi X, Gerard RD, Richardson JA (2006). The transcriptional coactivator CAMTA2 stimulates cardiac growth by opposing class II histone deacetylases. Cell.

[39] Martin M, Kettmann R, Dequiedt F (2007). Class IIa histone deacetylases: regulating the regulators. Oncogene.

[40] Qian M-X, Pang Y, Liu Cui H, Haratake K, Du B-Y, Ji D-Y (2013). Acetylation-mediated proteasomal degradation of core histones during DNA repair and spermatogenesis. Cell.

[41] Ustrell V, Hoffman L, Pratt G, Rechsteiner M (2002). PA200, a nuclear proteasome activator involved in DNA repair. EMBO J.

[42] Srinivas H, Xia D, Moore NL, Uray IP, Kim H, Ma L (2006). Akt phosphorylates and suppresses the transactivation of retinoic acid receptor alpha. Biochem J.

[43] Li XS, Trojer P, Matsumura T, Treisman JE, Tanese N (2010). Mammalian SWI/SNF-A subunit BAF250/ARID1 is an E3 ubiquitin ligase that targets histone H2B. Mol Cell Biol.

[44] Hoyer J, Ekici Arif B, Endele S, Popp B, Zweier C, Wiesener A (2012). Haploinsufficiency of ARID1B, a member of the SWI/SNF-A chromatin-remodeling complex, is a frequent cause of intellectual disability. Am J Hum Genet.

[45] Pause A, Lee S, Worrell RA, Chen DY, Burgess WH, Linehan WM (1997). The von Hippel-Lindau tumor-suppressor gene product forms a stable complex with human CUL-2, a member of the Cdc53 family of proteins. Proc Natl Acad Sci USA.

[46] Kamura T, Maenaka K, Kotoshiba S, Matsumoto M, Kohda D, Conaway RC (2004). VHL-box and SOCS-box domains determine binding specificity for Cul2-Rbx1 and Cul5-Rbx2 modules of ubiquitin ligases. Genes Dev.

[47] Forrester NA, Sedgwick GG, Thomas A, Blackford AN, Speiseder T, Dobner T (2011). Serotype-specific inactivation of the cellular DNA damage response during adenovirus infection. J Virol.

[48] Cukras S, Morffy N, Ohn T, Kee Y (2014). Inactivating UBE2M impacts the DNA damage response and genome integrity involving multiple cullin ligases. PLoS ONE.

[49] Zhao W, Huang Y, Zhang J, Liu M, Ji H, Wang C (2017). Polycomb group RING finger proteins 3/5 activate transcription via an interaction with the pluripotency factor Tex10 in embryonic stem cells. J Biol Chem.

[50] Duty SM, Singh NP, Silva MJ, Barr DB, Brock JW, Ryan L (2003). The relationship between environmental exposures to phthalates and DNA damage in human sperm using the neutral comet assay. Environ Health Perspect.

[51] Hauser R, Meeker JD, Singh NP, Silva MJ, Ryan L, Duty S (2006). DNA damage in human sperm is related to urinary levels of phthalate monoester and oxidative metabolites. Hum Reprod.

[52] Heebøll S, Borre M, Ottosen PD, Andersen CL, Mansilla F, Dyrskjøt L (2008). SMARCC1 expression is upregulated in prostate cancer and positively correlated with tumour recurrence and dedifferentiation. Histol Histopathol.

[53] McLay DW, Clarke HJ (2003). Remodelling the paternal chromatin at fertilization in mammals. Reproduction.

[54] Li J, Tsuprykov O, Yang X, Hocher B (2016). Paternal programming of offspring cardiometabolic diseases in later life. J Hypertens.

[55] Maalouf WE, Alberio R, Campbell KH (2008). Differential acetylation of histone H4 lysine during development of in vitro fertilized, cloned and parthenogenetically activated bovine embryos. Epigenetics.

[56] Lacagnina S (2020). The developmental origins of health and disease (DOHaD). Am J Lifestyle Med.

[57] Soubry A (2018). POHaD: why we should study future fathers. Environ Epigenet.

[58] Wu H, Estill MS, Shershebnev A, Suvorov A, Krawetz SA, Whitcomb BW (2017). Preconception urinary phthalate concentrations and sperm DNA methylation profiles among men undergoing IVF treatment: a cross-sectional study. Hum Reprod.

[59] Tian M, Liu L, Zhang J, Huang Q, Shen H (2019). Positive association of low-level environmental phthalate exposure with sperm motility was mediated by DNA methylation: a pilot study. Chemosphere.

[60] Zheng H, Zhou X, Li D-k, Yang F, Pan H, Li T (2017). Genome-wide alteration in DNA hydroxymethylation in the sperm from bisphenol A-exposed men. PLoS ONE.

[61] Oluwayiose OA, Marcho C, Wu H, Houle E, Krawetz SA, Suvorov A (2021). Paternal preconception phthalate exposure alters sperm methylome and embryonic programming. Environ Int.

[62] Virant-Klun I, Leicht S, Hughes C, Krijgsveld J (2016). Identification of maturation-specific proteins by single-cell proteomics of human oocytes. Mol Cell Proteomics.

[63] Castillo J, Jodar M, Oliva R (2018). The contribution of human sperm proteins to the development and epigenome of the preimplantation embryo. Hum Reprod Update.

[64] Smith CL, Eppig JT (2009). The mammalian phenotype ontology: enabling robust annotation and comparative analysis. Wiley Interdiscip Rev Syst Biol Med.

[65] Kent WJ, Sugnet CW, Furey TS, Roskin KM, Pringle TH, Zahler AM (2002). The human genome browser at UCSC. Genome Res.

[66] Kuleshov MV, Jones MR, Rouillard AD, Fernandez NF, Duan Q, Wang Z (2016). Enrichr: a comprehensive gene set enrichment analysis web server 2016 update. Nucleic Acids Res.

[67] Chen EY, Tan CM, Kou Y, Duan Q, Wang Z, Meirelles GV (2013). Enrichr: interactive and collaborative HTML5 gene list enrichment analysis tool. BMC Bioinformatics.

[68] Stelzer G, Rosen N, Plaschkes I, Zimmerman S, Twik M, Fishilevich S (2016). The GeneCards suite: from gene data mining to disease genome sequence analyses. Curr Protoc Bioinformatics.

