# Supplementary figures and images for: Phthalates impact on the epigenetic factors contributed specifically by the father at fertilization
Source: Epigenetics Chromatin. 2023 Jan 24;16:3. doi: 10.1186/s13072-022-00475-2 (PMC9872317; doi:10.1186/s13072-022-00475-2)

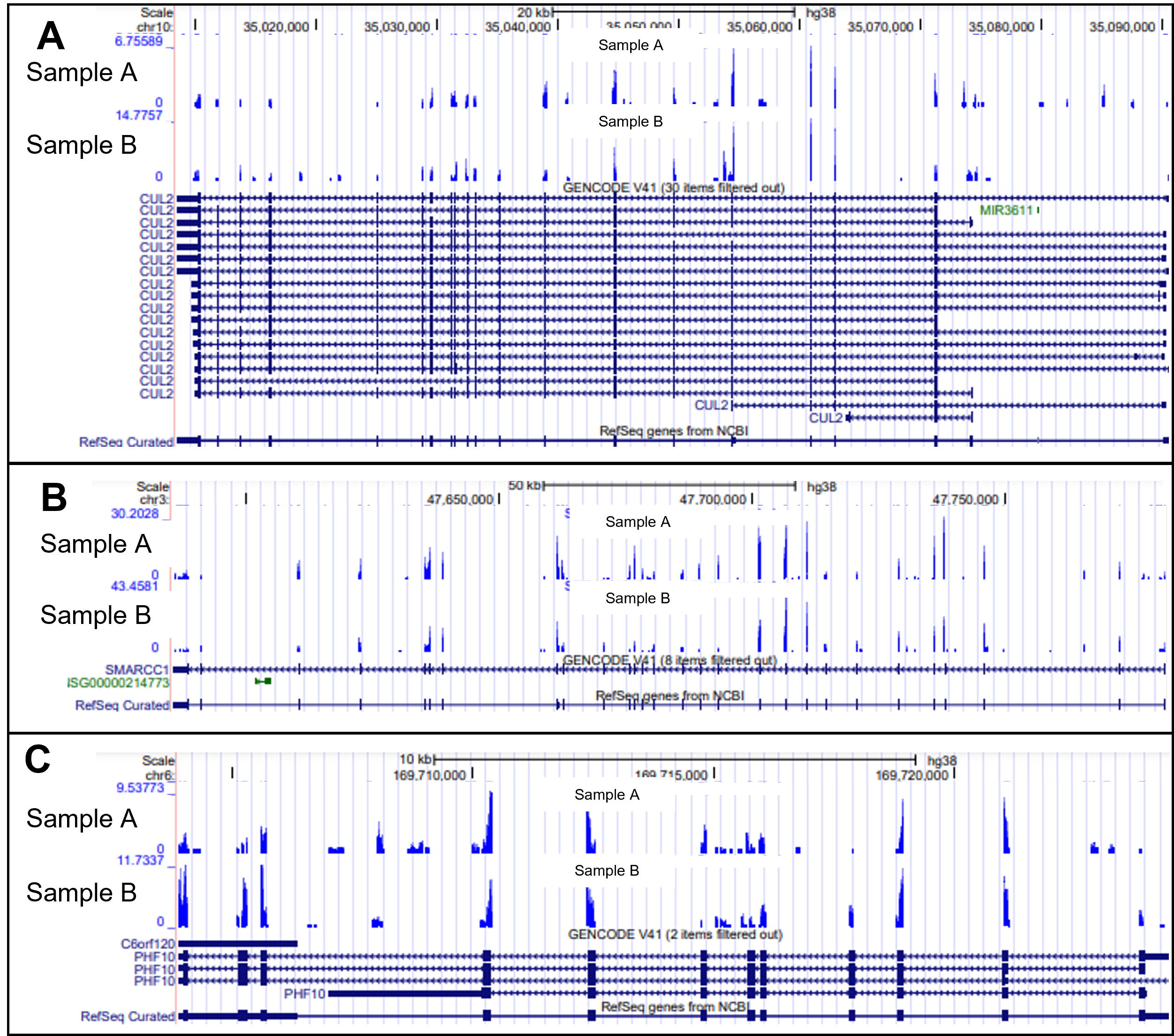

Supplement: Supplementary file 1 — Additional file 1: Figure S1. Chromatin remodeler cofactor, RNA interactor, reader, eraser and writer (CRREW) RNA Element (RE)-containing RNA (RE-RNA) visual integrity. Representative samples chosen for A) CUL2, B) SMARCC1 and C) PHF10. Integrity of DBP responsive and paternally provided CRREWs was determined using the UCSC Genome Browser Gencode version 41 track. Threshold for an RE-RNA to be considered full-length was set at a minimum of 5 Reads per Kilobase per Million (RPKM) across all transcript exons in all 7 paternally provided samples and all DBP responsive samples (high-DBP study arm (H1BH2), 55 samples; background-DBP study (B1HB2) arm: 35 samples). [file 13072_2022_475_MOESM1_ESM.png]
